# Massachusetts Veterinary Association

**Published:** 1892-08

**Authors:** Austin Peters

**Affiliations:** Sec’y.


*Massachusetts Veterinary Association:*—The Regular Meeting of the Massachusetts Veterinary Association, was held at 19 Boylston Place, Boston, Wednesday evening, June 22, 1892, at 7.30 o'clock. The President, L. H. Howard, in the chair. Members present: Drs. Bryden, Blackwood, Becket, Burr, Emerson, Hadcock, Harrington, Howard, Marshall, Osgood, Peterson, Winchester, Winslow, Hitchcock, Parker, LaBaw and the Secretary. Honorary member: Dr. Stickney. Guests: Mr. T. L. Bolton, of Clark University, essayist of the evening; E. P. McKenna, M. D. V., and Mr. W. M. Balmer.

The records of the last meeting were read and accepted.

Unfinished business: Motion made by Dr. Hitchcock that Dr. Bunker be given a vote of thanks for the paper read by him at the last meeting. Seconded by Dr. Emerson. Carried.

Dr. Burr brought up the point that cases reported by members should not be fathered by the Association as correct, unless the diagnosis was verified by a microscopic examination, in instance where the microscope could settle a matter of doubt. For instance, Dr. Bunker's case of tuberculosis of the liver in a bull should be settled by finding the tubercle bacilli in the specimen before we accept it as such. The members present seemed to think this point well taken. Dr. Howard here left the chair, which was taken by First Vice-President Burr. Dr. Burr thought it ought to be voted by the Association that it could not accept Dr. Bunker's case of tuberculosis of the liver until it was properly verified. Dr. Hitchcock thought it would be sufficient if the President ordered the Secretary to make a note in the records upon the point in dispute.

Admission of members: The name of Dr. Wm. Stinson, of Chelsea, a graduate of the New York College of Veterinary Surgeons, was then balloted upon, Dr. Winchester Teller. Sixteen votes were cast, all in the affirmative. Dr. Stinson was therefore declared elected.

Applications for membership: Applications for membership were received from Wilbert Sould, M.D.V., and E. P. McKenna, M.D.V. Dr. Osgood, Chairman of the Executive Committee, reported favorably upon their credentials. Their names are laid on the table, to be voted upon at the next regular meeting, according to the provisions of the Constitution.

Dr. Howard here took the chair again. He said that as this was the last meeting of our Association before the meeting of the United States Veterinary Medical Association in September, he thinks we ought to send its Secretary an official notice of our plans for entertaining its members.

Dr. Osgood makes the motion that we invite the members of the U.S.V.M.A. to be our guests Wednesday, the second day of the meeting. Seconded by Dr. Peterson. Dr Osgood amends his motion to read, Wednesday afternoon. Considerable discussion followed. Dr. Bryden opposed having a recess the second day, on the ground that our entertainment would break in too much on the routine work laid out for the meeting. Dr. Winchester thought that if a recess were taken by three or four o'clock Wednesday afternoon, it could do no harm. Drs. Osgood and Hitchcock both spoke in favor of breaking the monotony of the meeting by having our Association's entertainment upon the afternoon of the second day, Dr. Burr sided with Dr. Bryden, believing we should do nothing to interfere with the routine of the meeting, and that it would be better for our entertainment to come upon the third day.

The question was then put, and the motion was carried.

Mr. T. L. Bolton, of Clark University, at Worcester, then read a very interesting paper upon the results of the microscopic examinations made there upon the spinal cord of the old horse killed at Ward's Wharf last July, owned by the Association, and treated awhile by Dr. Bryden. (For Dr. Bryden's report see the records of the meeting held November 25, 1891). Mr. Bolton's paper proved very interesting. He reported certain degenerative changes in portions of the cord and its nerves sent to the University for examination. Of course one swallow does not make a summer, but it would be very interesting to examine a number of cords from horses with springhalt, and see if similar changes could be found.

(Note: Members of the veterinary profession having any cases of death among their patients that may be afflicted with springhalt, might be benefiting science by removing the portion of the cord, with some of the nerves of the lumbo-sacral plexus, and sending the same to Dr. H. H. Donaldson or Mr. T. L. Bolton, Clark University, Worcester, Mass., where they will be very glad to make the microscopic examination. If possible, a history of the case ought to accompany the specimen. The specimen should contain the cord posterior to the tenth dorsal vertebra, with some of the lumbo-sacral nerves. It should be carefully removed, and sent in a glass jar containing a two and a half per cent. solution of bichromate of potash plus one-sixth of its volume of ninety-five per cent. alcohol.)

Mr. Bolton's paper was discussed by many of the members present. Dr. Bryden said that he took cognizance of the fact that there might be changes in the spinal cord, but he questioned whether they were primary or secondary. He holds that solipeds, animals wlth feet confined in a horny box, are liable to peculiar forms of lameness, and that these troubles begin in the hoof, leading later to disturbances higher up; these might occur in the muscles first, causing the tail to be carried to one side, or skipping a little when hurried, before going lame. So in navicular disease, the first changes take place in the horny box itself, afterward leading to the changes in the bone, etc. Dr. Bryden believes in "Hoof Culture." Don't fire and blister a spavin—it is humiliating to the profession—but attend to the foot. But to return to this case, he thinks the changes in the cord were secondary.

Mr. Bolton does not agree to this, because sensory changes in the cord cannot be secondary. The degeneration is *from* the *centre* to the *periphery*. Dr Bryden thinks that solipeds have a class of diseases that no other animal is subjected to, and that these progress from the periphery in, and cannot be a chorea or anything like that. He thinks we have a splendid subject for investigation, but holds that it is peripheral disturbance. Dr. LaBaw thinks the breaking of a small artery anterior to the lesion found ought not to cause a degeneration of the cord, as a small artery is given off at every articulation, anastomoses are free there. But, Mr. Bolton says, if a nerve's nutritive supply is cut off, that fibre dies to where it comes in contact with the next nerve cell, the nerve cell is the centre of nutrition for that fibre. That is, for a sensory fibre, while a motor fibre degenerates toward the brain.

Dr. Marshall wants to know if it can be an hereditary disease. Can a blood vessel be hereditarily weakened? All do not agree that it is hereditary. Dr. Parker remembered seeing a tumor in a cord, in which case degeneration extended both ways. Can this occur from the rupture of a blood vessel? Mr. Bolton: "That depends upon the size of the area supplied by that blood vessel."

Motion made by Dr. Hitchcock, that we extend to Mr. Bolton and the professors of Clark University, a vote of thanks for the trouble they have taken in investigating this case. Seconded by Dr. LaBaw. Carried enthusiastically.

Moved by Dr. Hadock that we have a special meeting the second Wednesday in September. Seconded by Dr. Emerson. Carried.

Meeting then adjourned.

AUSTIN PETERS, *Sec'y.*

